# Endoscopic and Endoscopically-Assisted Resection of Intraventricular Lesions Using a Neuroendoscopic Ultrasonic Aspirator

**DOI:** 10.3390/jcm10173889

**Published:** 2021-08-29

**Authors:** Florian Ebel, Ladina Greuter, Maria Licci, Raphael Guzman, Jehuda Soleman

**Affiliations:** 1 Department of Neurosurgery, University Hospital of Basel, 4031 Basel, Switzerland; ladina.greuter@usb.ch (L.G.); maria.licci@usb.ch (M.L.); raphael.guzman@usb.ch (R.G.); jehuda.soleman@gmail.com (J.S.); 2 Division of Pediatric Neurosurgery, University Children’s Hospital of Basel, 4056 Basel, Switzerland; 3 Faculty of Medicine, University of Basel, 4001 Basel, Switzerland

**Keywords:** neuroendoscopy, ultrasonic aspirator, surgical technique

## Abstract

The development of minimally invasive neuroendoscopy has advanced in recent years. The introduction of the neuroendoscopic ultrasonic aspirator (NUA) broadened the treatment spectrum of neuroendoscopy. We aim to describe our experience with the use of NUA for the resection of intraventricular lesions. Here, we present consecutive retrospective case series of adult and pediatric patients undergoing resection of an intraventricular lesion with a NUA (Endoscopic Neurosurgical Pen, Söring GmbH, Quickborn, Germany) between January 2019 and April 2020. Eight patients between the age of 0.5 and 73 years underwent surgery using NUA and were included in this study. In four patients, an endoscopic assisted (EA) resection of the lesion was undertaken, while in four patients, the lesion was removed using purely endoscopic (PE) resection. In all cases, gross/near total resection was achieved. The average blood loss was 142.5 ± 90.4 mL (range 50–300 mL). Transient morbidity was seen in four patients (50%), while permanent morbidity or mortality did not occur. The NUA seems to be a safe and valuable tool for the minimally invasive resection of intraventricular lesions in selected cases. The type, size, consistency, and vascularization of the lesion limit at times the purely endoscopic use of the NUA.

## 1. Introduction

Neuroendoscopy has increasingly become a valuable instrument for the treatment of various neurosurgical pathologies in recent decades [1]. Prior to the introduction of neuroendoscopic ultrasonic aspirators (NUA), the fields of application for neuroendoscopy were limited to the treatment of hydrocephalus, performance of biopsies, or partial resection of brain lesions [2]. In 2008, the use of an NUA was described for the first time [2]. Since then, only few studies describing the use of the NUA have been published, pointing at the limited experience available and reported in this field [2,3,4,5,6,7]. This study aims to describe our experience of the purely endoscopic and endoscopically assisted resection of lesions using NUA.

## 2. Materials and Methods

### 2.1. Patient Population

The study involved consecutive retrospective single-center case series of adult and pediatric patients undergoing resection of an intraventricular lesion with a NUA (Endoscopic Neurosurgical Pen, Söring GmbH, Quickborn, Germany) between January 2019 and April 2020 at the Department of Neurosurgery and Division of Pediatric Neurosurgery University Hospital of Basel, Switzerland. Data were extracted from our surgical logbook and the patients’ medical files. Baseline characteristics such as age, gender, clinical symptoms, presence of hydrocephalus or papilledema, location and size (diameter and volume) of the lesion, complementary use of microscope in terms of an endoscopic-assisted technique, and type of surgical approach were collected. We then analyzed the extent of resection (gross total/near total resection or subtotal resection), surgery time, intraoperative blood loss, histological diagnosis, intraoperative and postoperative complications (transient/permanent) with corresponding necessary follow-up operations, neuropsychological evaluation, modified Rankin Scale (mRS), improvement of clinical symptoms (no symptoms, improved, stable, worsened), mean follow-up time, and the recurrence of the lesion. Neuropsychological evaluation using the Montreal Cognitive Assessment score (MOCA; the maximum possible score is 30 points with a score of <26 considered as having neurocognitive impairment [8]) was carried out if there was a subjective impression of a new neurocognitive deficit postoperatively. The extent of resection was always assessed by a board-certified neuroradiologist based on postoperative T1-weighted MRI imaging sequences with and without contrast, which was performed within 48 h postoperatively. Based on the neuroradiological assessment, we defined gross total resection (GTR) as a complete resection of the mass without evidence of a residual on postoperative imaging. Near total resection (NTR) was defined as a small contrast enhancing residual in the resection cavity area. If larger remnants were obviously left in situ during surgery, we assessed them as subtotal resection (STR). Regarding the extent of resection in colloid cysts, a recently published study has shown that colloid cyst resection without complete removal of the cyst wall is equivalent to complete removal of the cyst wall in terms of long-term recurrence rate and outcome [9]. Therefore, we deliberately combined NTR and GTR into one group, since we achieved NTR only in one patient suffering from a colloid cyst. The data are presented descriptively, providing mean, standard deviation (SD), and range. The study protocol was approved by the local ethics committee (EKNZ, Basel, Switzerland), whereby patient consent was waived.

### 2.2. Surgical Procedure

**Figure 1:**
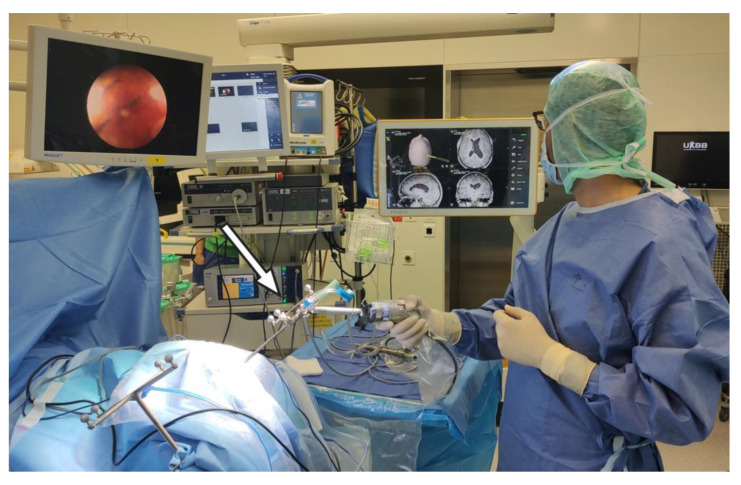
Setup in the OR with the endoscopy tower on the left side of the picture and the navigation system’s screen on the right side. The endoscope is linked to the navigation system (arrow).

**Figure 2:**
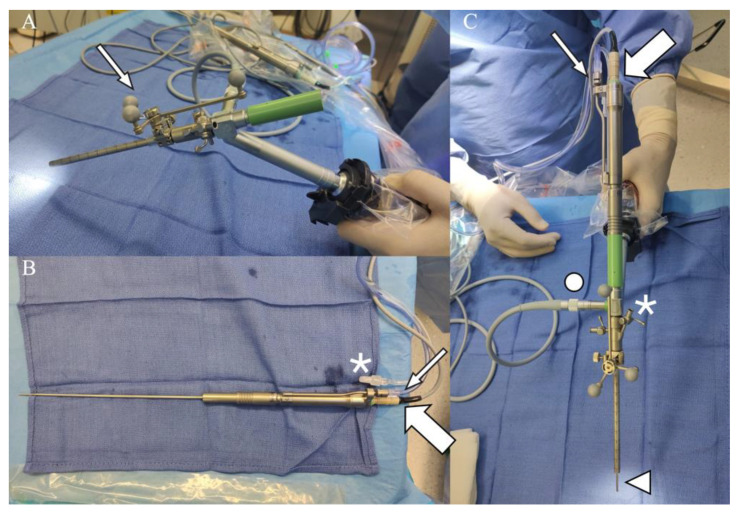
(**A**) 0° Gaab rigid endoscope (Karl Storz GmbH, Tuttlingen, Germany) with the attached three-point reference for navigation (arrow). (**B**) The NUA tool is presented (Endoscopic Neurosurgical Pen, Söring GmbH, Quickborn, Germany). The connection site of the aspiration (thin arrow) and ultrasonic oscillator (thick arrow) of the UA SYSTEM with the NUA are presented. The irrigation (asterisk) is connected directly to the endoscope. (**C**) Presenting the two instruments (NUA pen inserted into the GAAB endoscope) combined. The connections with the aspiration (thin arrow) and ultrasonic oscillating (thick arrow) system of the UA and the site (asterisk) where the irrigation is connected to the endoscope are presented. The NUA pen tip emerging through the endoscope (triangle), and the light source (circuit) and attached three-point reference for navigation are presented.

As of 2019, in our institution, the usage of NUA was commenced. All patients with intraventricular lesions referred to us in the period mentioned above were treated with the newly introduced NUA. There were no purely intraventricular lesions treated primarily by conventional open microsurgery during this period. All patients received single shot antibiotic (Cefuroxim) half an hour before incision, and surgery was completed under general anesthesia. The head was fixed in a Mayfield head clamp (Integra LifeSciences Corp., Plainsboro, NJ, USA) and Neuronavigation (Brainlab AG, München, Germany) was used. For better anatomical intraventricular orientation, the navigation was linked to the endoscope in all cases (Figure 1). All lesions expected to be either vascularized, of solid or calcified consistency, or larger than 2 cm in diameter, the surgical approach was tailored for a possible conversion to microscopic surgery (Table 1). In such cases, a mini-craniotomy of approximately 2 × 2 cm and introduction of a brain speculum (Vycor, Vycor Medical Inc., Boca Raton, FL, USA) were concluded, while the operation was commenced endoscopically and only if the needed conversion to a microscopical resection was done. Finally, in the four patients who underwent mini-craniotomy due to the aforementioned lesions’ characteristics, conversion to microscopic resection was made intraoperatively in all cases due to the high consistency (cases no. 1 and 3), the high vascularization (cases no. 6 and 7), or the size (case no. 7) of the tumor. All other lesions were approached purely endoscopically through a frontal burr hole using the Kocher’s standard entry point with introduction of a peel-away sheath. A 0° Gaab rigid endoscope (Karl Storz GmbH, Tuttlingen, Germany) and a NUA were used to resect the lesion. The OR setup, equipment, and resection of an intraventricular lesion are shown in the supplementary Video as well as in Figure 1 and Figure 2. All patients were operated by the senior authors (R.G. and J.S.).

## 3. Results

### 3.1. Baseline Characteristics

Eight consecutive patients, of whom five were males (62.5%), with a mean age of 41.7 ± 28.2 years (range 0.5–73 years) were included. The cohort consisted of three pediatric (37.5%; range 6 months–16 years) and five adult patients (62.5%; range 42–73 years; Table 2). Depending on the above-mentioned aspects of the target lesion, in four patients (50%), an endoscopically assisted (EA) approach and in four patients (50%), a purely endoscopic (PE) approach was finally performed. A frontal approach was chosen in five cases (62.5%), an occipital approach in two cases (25%), and a parietal approach in one case (12.5%). The maximum diameter and the volume of the lesion mass were on average 24.1 ± 12.9 mm (range 9–45 mm) and 7.09 ± 8.6 cm^3^ (range 0.2–23.8 cm^3^), respectively (Table 2).

### 3.2. Outcome Measurements

**Figure 3:**
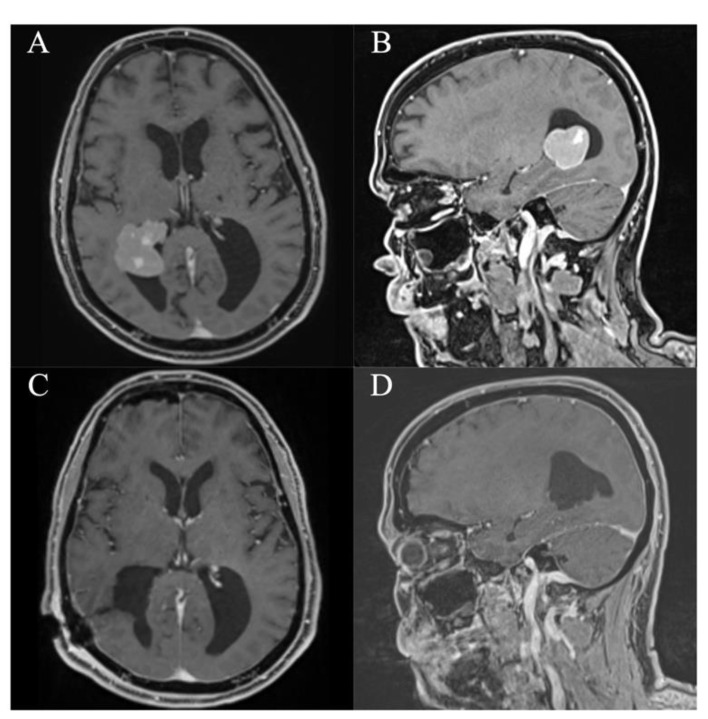
Case 3. Axial and sagittal MR images, T1-weighted sequences with contrast medium preoperatively (**A**,**B**) and immediately postoperatively (**C**,**D**) after resection of the intraventricular meningeoma via a right parietal endoscopic-assisted approach.

**Figure 4:**
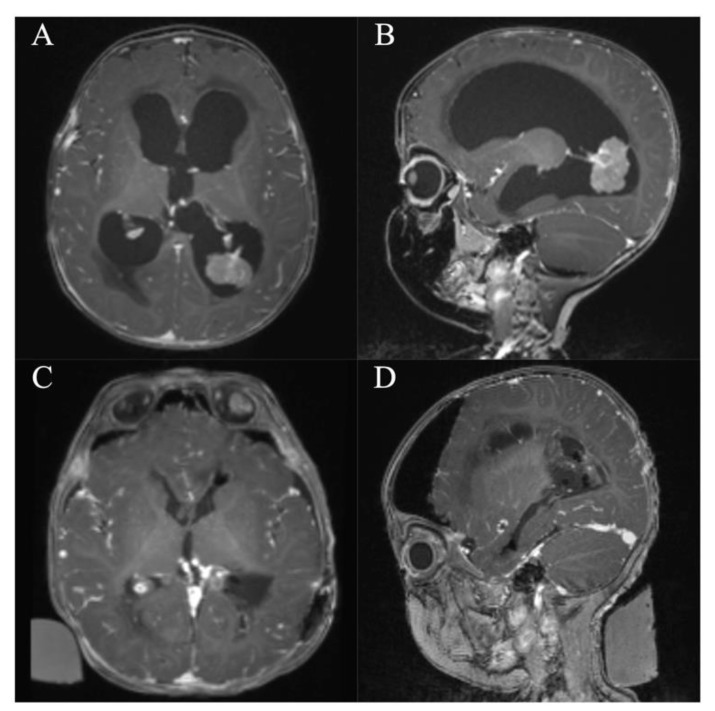
Case 6. Axial and sagittal MR images, T1-weighted sequences with contrast medium preoperatively (**A**,**B**) and immediately postoperatively (**C**,**D**) after resection of the intraventricular choroid plexus carcinoma via a left occipital endoscopic-assisted approach.

**Figure 5:**
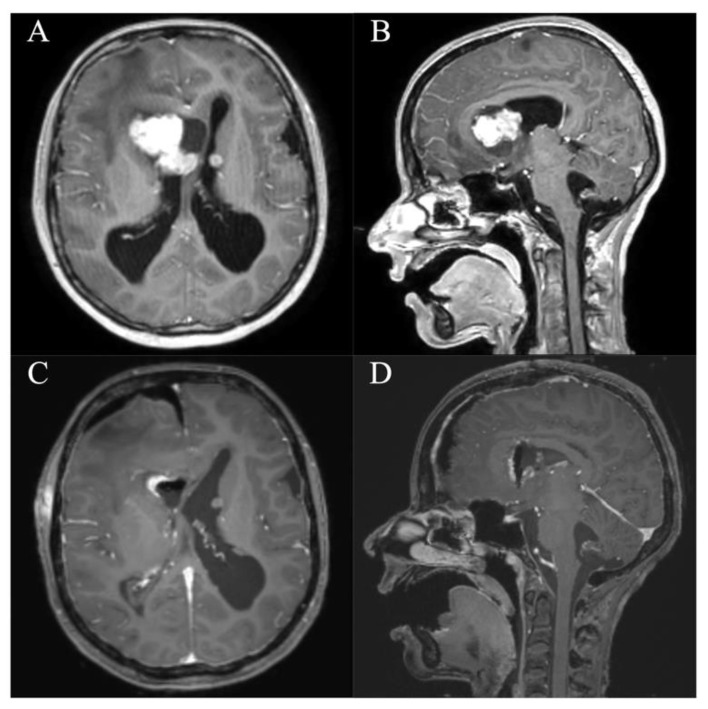
Case 7. Axial and sagittal MR images, T1-weighted sequences with contrast medium preoperatively (**A**,**B**) and immediately postoperatively (**C**,**D**) after resection of the intraventricular subependymal giant cell astrocytoma via a right frontal endoscopic-assisted approach. It shows a gross total resection with residual blood in the resection cavity. Furthermore, there are pre-existing subependymal tuberosities in both lateral ventricles.

**Figure 6:**
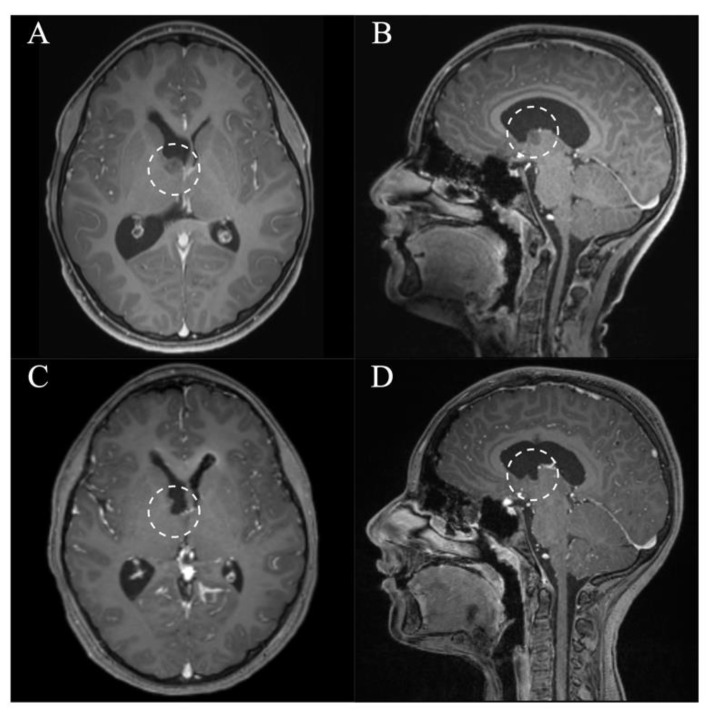
Case 8. Axial and sagittal MR images, T1-weighted sequences with contrast medium preoperatively (**A**,**B**) and immediately postoperatively (**C**,**D**) after resection of a lesion originating from the right anterior thalamus via a right frontal purely endoscopic approach. It shows a gross total resection. The lesion and resection cavity is marked with a dotted circle.

In all patients, a GTR/NTR could be achieved (Table 3). The pre- and postoperative scans of cases no. 3, 6, and 7, which were all treated through an EA approach and case no. 8, which was treated through a PE approach, are shown in Figure 3, Figure 4, Figure 5 and Figure 6. In one patient (case no. 5), due to her advanced age, we referred from resecting the vascularized capsule of the colloid cyst, achieving NTR. The mean surgery time was 163.6 ± 54.2 min (range 82–240 min) and the average blood loss was 142.5 ± 90.4 mL (range 50–300 mL). The PE approach was associated with lower mean blood loss than the EA approach (87.5 ± 47.8 mL vs. 197.5 ± 93.2 mL, *p* = 0.114). In four patients (50%), a total of six postoperative complications occurred, of which all were transient. Two patients (25%) showed transient cognitive impairments after resection of a colloid cyst, most likely directly associated with the endoscopic approach. The remaining four transient complications were unrelated to the use of endoscopy or NUA (Table 3). The two patients with neuropsychological evaluation showed postoperatively scores of 18/30 and 25/30 points, respectively, with a documented improvement in both patients at follow-up (18/30 to 24/30 points within 35 days and 25/30 to 30/30 points within 114 days). With regards to the remaining adult patients, no evaluation was carried out, because there was no evidence of neurocognitive deficits subjectively. In the two children with intraventricular lesions, no postoperative neurocognitive assessment was performed (due to autism disorder in the context of tuberous sclerosis in one case and due to the very young age of 5 months in the other case) (Table 3). At follow-up (15.9 ± 6.3 months; range 6.8 to 23.2 months), all patients showed improved or unchanged mRS when compared to the mRS at discharge. Complete regression or improvement of the preoperative complaints was seen in all patients, while MRI at follow-up showed no recurrence in any of the cases (Table 3).

## 4. Discussion

We present a single-center case series consisting of eight patients with intraventricular lesions resected purely endoscopically or endoscopically assisted using a NUA. Based on our experience, small lesions under the size of 2 cm can be resected safely with the PE technique. Lesions larger than 2 cm present a greater challenge for PE resection, especially if these lesions are suspected to be of solid consistency (e.g., intraventricular meningiomas) or highly vascularized (e.g., CPC). Clearly, a learning curve for the technically challenging use of the NUA exists, thus not excluding the successful resection of larger lesions with increasing experience. To this purpose, the development of teaching models for endoscopy and NUA use additionally supports the training without the involvement of patients [10]. So far, our recommendation would be to tailor the approach (burr hole vs. mini-craniotomy in combination with a speculum) according to the surgeon’s experience, the size of the lesion, and its characteristics (Table 1). Based on our results, four patients (50%) had a total of six postoperative complications, all of which were transient and four of which were not directly associated with the endoscopic approach nor the use of NUA. However, permanent morbidity seems rare, and based on the aforementioned lesions’ characteristics with the appropriate endoscopic approach and the surgeon’s experience, GTR can be safely achieved in most cases.

### 4.1. Advantages Compared to Conventional Neuroendoscopic Instruments

Conventional neuroendoscopic instruments for resecting intraventricular lesions have included, so far, the suction device, grasping forceps, and the dissector [11,12,13]. With these instruments, it was possible to partially or entirely resect soft and small intraventricular tumors in technically challenging and time-consuming procedures. According to a large meta-analysis conducted by Barber et al., which focused on the endoscopic resection of intraventricular tumors, GTR/NTR could be achieved in 75% of the cases [1]. In most of the studies examined within this meta-analysis, only those standard endoscopic instruments were used, while only in two cases an aspiration tool based on the NUA technology was deployed [1].

Ultrasound aspirators have become widely established to remove tumors in open microsurgery, whereupon the endoscopic use of an NUA was first described in 2008 [2]. At about the same time, another tool, the NICO Myriad System (NICO Corp., Indianapolis, IN, USA), was developed. This is a purely mechanical side-cutting aspiration system. The use of this system has been described in the literature with satisfactory results [14,15].

Both systems have opened up new dimensions in neuroendoscopic removal of intraventricular lesions through their highly efficient functioning, as they combine several functions such as dissector, aspirator, and tissue cutter in one tool. The mechanisms of these tools are different, although we assume that the NUA and its adjustable power of the tip’s vibration make tissue selection possible, as we also know from open microsurgery, thus enabling a gentler resection, especially the sparing of the vessels. Tissue selection based on their structure is not possible with the NICO Myriad System (NICO Corp., Indianapolis, IN, USA) due to its purely mechanical cutting function.

In the largest case series to date, including 12 cases in which intra- and paraventricular brain lesions were resected purely endoscopically using NUA, a GTR/NTR was achieved in 75% of cases [3]. In our series, a GTR/NTR could be achieved in all patients. Overall, we are convinced of the technique and efficiency of the NUA for the resection of intraventricular lesions and think that with our series, we contribute to emphasize further the advantages of the NUA in the removal of these lesions. However, the indication remains an important factor influencing the resection rate and outcome.

### 4.2. Factors Indicating the Endoscopic Approach

The average size of lesions operated with the use of NUA reported in the literature is 20.6 ± 9.7 mm (range 11–42 mm) [4]. In our series, the average size was higher with a mean of 24.1 ± 12.9 mm (range 9–45 mm). This might explain why in four of the eight patients in our series, the initially planned PE resection was converted to an EA resection. Nevertheless, apart from the lesion’s size, our experience indicates that further lesional factors, such as vascularity, consistency, and attachment to adjacent structures, seem to affect whether the lesion can be removed through a PE approach (Table 1). Selvanathan et al. first described the use of NUA for neuroendoscopic resection of a solid low-grade glioneuronal tumor, located within the aqueduct. Subsequently, in the two largest case series, a total of 21 lesions were removed with a PE approach by utilizing the NUA [3,4]. The most frequent lesions were colloid cysts (*n* = 3, 14.3%), pilocytic astrocytoma (*n* = 3, 14.3%), subependymoma (*n* = 3, 14.3%), followed by SEGA (*n* = 2, 9.5%), low-grade glioneuronal tumor (*n* = 2, 9.5%), craniopharyngioma (*n* = 2, 9.5%), medulloblastoma (*n* = 1, 4.8%), epidermoid tumor (*n* = 1, 4.8%), central neurocytoma (*n* = 1, 4.8%), pineal anlage tumor (*n* = 1, 4.8%), atypical teratoid rhabdoid tumor (*n* = 1, 4.8%), and immature teratoma (*n* = 1, 4.8%) [3,4]. In general, based on the current literature, the NUA is recommended for the removal of soft lesions with poor vascularization and a size of less than 2–3 cm [3,4,16,17].

Based on our experience, when resecting lesions endoscopically, we converted to microscopic resection for the following reasons: 1. In case of very vascularized lesions (the CPC and SEGA case, no. 6 and no. 7); 2. Calcified or solid lesions were the tip of the NUA was too small and delicate to achieve sufficient resection in an adequate time, even with a cavitation intensity setting of 80% (the meningeoma cases, no. 1 and no. 3); and 3. Large lesions, occupying a vast space within the ventricle with extensive attachment to the ependyma causing bleeding and visual impairment (the SEGA case, no. 7). However, we do believe that due to the rather steep learning curve that we experienced in resecting lesions using NUA through a PE approach, with further experience, the safe and efficient resection of large, solid, and vascularized lesions will be possible.

### 4.3. Advantages and Disadvantages of a Pure Endoscopic vs. an Endoscopic-Assisted Approach

In our case series, we tried in principle to perform a purely endoscopic approach in all cases. Due to the factors and circumstances mentioned above, 50% of the lesions were finally resected using an endoscopic-assisted technique.

**Figure 7:**
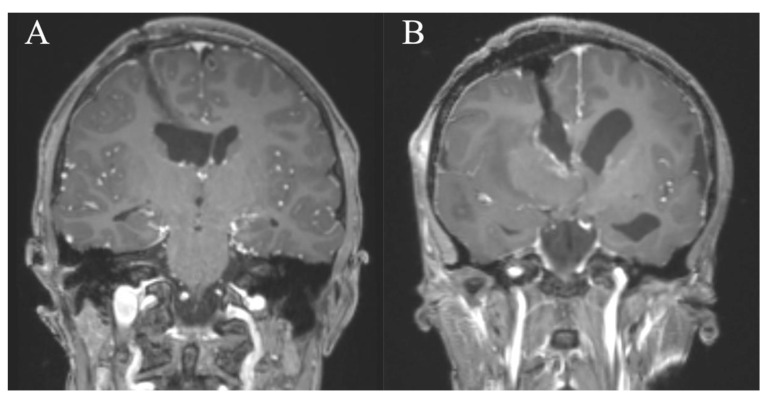
Cases 8 and 7. Coronal MR images, T1-weighted sequences with contrast medium immediately postoperatively (**A**,**B**) after resection of an intraventricular lesion through a frontal right approach in a pure endoscopic (**A**) and endoscopic-assisted fashion (**B**). A PE approach tends to result in less tissue damage to the cortex and parenchyma due to the small diameter peel-away sheath (**A**) compared to the endoscopic-assisted approach using the Vycor speculum (**B**).

In contrast to the EA technique, the PE technique involves only a very small skin incision, a burr hole, and small corticotomy which results in minimal tissue damage (Figure 7). We hypothesize that the minimally invasive approach of the PE technique and the associated minimal tissue damage will reduce the risk of wound healing problems, CSF fistulas, parenchymal bleeding, and further surgery-related complications compared to the EA technique.

In comparison, the range of motion of the endoscope through a PE approach is limited. Using an EA approach with a mini-craniotomy, the range of motion and the viewing angles with the endoscope is wider, and there is better control in the event of bleeding. In addition, an EA technique can be used to remove large parts of a lesion using the microscope, if needed. The resection of the lesion can then be done using both the microscope and the endoscope, which might be essential for lesions with characteristics as descried in Table 1.

The above-mentioned aspects are merely subjective experiences, and conclusive recommendations are currently not possible due to the lack of evidence. Larger case series or randomized studies are needed.

### 4.4. Perioperative Complications

Intra- and postoperative complication rates of the PE NUA approach are reported between 0% and 66.7% [2,3,4]. In the series from Cinalli et al., a complication rate of 8.3% is reported [3]. Ibáñez-Botella et al. reported two (22.2%) intraoperative and six (66.7%) postoperative complications, while in two patients, an EVD was inserted due to rather extensive bleeding during tumor resection [4]. Oertel et al. showed no intra- or postoperative complications in their series [2]. In our cohort, two intraoperative (25%) and six (75%) postoperative complications occurred, of which all were transient. According to our data, the most frequent complication was transient cognitive impairment (*n* = 3, 33.3%). A comparative statement to other studies is not possible, since the postoperative cognitive outcome was not assessed in any of the studies mentioned above. Generally, based on a recently published systematic review analyzing cognitive outcomes after neuroendoscopic procedures, these outcomes are often underreported [18]. It is known that regardless of the use of NUA, the most common neurocognitive complication after ventricular neuroendoscopy is memory impairment (2% transient and 1% permanent memory impairment). Specifically, for the resection of a colloid cyst, it is significantly higher, accounting for 7.96% and 2.65%, respectively [18].

### 4.5. Management of Intraoperative Hemorrhage

Intraoperative bleeding from the lesion or the ependyma attached to the lesion occurred in one case within our cohort. In endoscopic surgery, vision can be severely restricted even by slight bleeding. Based on our experience and the existing literature, various techniques for hemorrhage control exist, including excessive rinsing, monopolar coagulation, using the heat intensity provided by the light of the endoscope, creating a “fluid chamber” using the trocar or peel-away sheath (“small-chamber irrigation technique”), or using the dry field technique by sucking the CSF out of the ventricles to identify and coagulate the source of bleeding and the air environment also supports the clot formation [19,20,21,22]. These techniques might not suffice for very vascularized lesions compared to microscopic surgery techniques, since forceps for coagulation and adequate suction are not available for neuroendoscopic surgery. Therefore, the surgeon should always tailor the approach (burr-hole vs. mini-craniotomy and speculum) according to the type of lesion and expected difficulty, while he or she should always be prepared to convert to microscopic surgery. In our experience, the rinsing function of the Gaab endoscope is less effective as opposed to the Lotta endoscope (Karl Storz GmbH, Tuttlingen, Germany). This is most probably due to a different construction of the endoscope tip. While the Lotta endoscope tip is circular, it produces a more centered water jet, which provides more focused rinsing of the bleeding and better visualization. The Gaab endoscope, on the other hand, is crescent-shaped, leading to a less centered jet of water, making the rinsing of the bleeding focus less effective. To date, the NUA provided from Söring is compatible only with a Gaab endoscope.

### 4.6. Limitations

Our study’s primary goal was to describe our technique and outcome; hence, this study is limited by its descriptive nature. First, no control group was analyzed within this cohort; therefore, firm conclusions on superiority or inferiority of the NUA technique compared to other techniques cannot be made. Second, our study includes only eight patients. Larger series are certainly needed to confirm our observations, and more extensive series comparing the NUA to the traditional microsurgical resection of lesions should be sought. Third, the neurocognitive examination was recorded inconsistently, which is why a conclusive statement regarding the effects of neuroendoscopy on neurocognition is not possible. Finally, the development of innovative techniques and tools improving the neuroendoscopic handling of lesion resection and intraoperative bleeding would surely broaden the indication for NUA usage.

## 5. Conclusions

Based on our presented case series, the NUA seems a safe and valid tool for the resection of intraventricular lesions. Complications after the resection of lesions using the NUA are usually transient, while based on the lesions’ characteristics with the appropriate endoscopic approach and the surgeon’s experience, GTR can be safely achieved in most cases. Purely endoscopic resection of a lesion using the NUA seems ideal for soft, minimally vascularized, and rather small (<2 cm) lesions. For large, vascularized, and solid or calcified lesions, depending on the surgeon’s experience with the NUA, preparing for a combined endoscopic and microscopic approach seems advisable.

## Figures and Tables

**Table 1:** Based on our experience, preoperatively suspected lesion characteristics favor either a purely endoscopic or an endoscopically assisted approach.

	Purely Endoscopic Approach	Endoscopically Assisted Approach
Size	<2 cm	>2 cm
Vascularization	Low	High
Consistency	Soft	Solid, calcified
Architecture	Simple (round, oval)	Complex (several compartments)

**Table 2:** Baseline characteristics of our case series.

No.	Gender	Age (Y)	Clinical Signs	Papilledema	Hydrocephalus	Histology	Lesion Location	D (mm)	V (cm^3^)	Approach
1	f	68	Headache	N/A	No	Meningeoma (WHO I)	Lateral ventricle left	26	5.9	Parietal left
2	m	59	Headache	Yes	Yes	Colloid cyst	Third ventricle	16	1.8	Frontal right
3	m	62	Headache	N/A	Yes	Meningeoma (WHO I)	Lateral ventricle right	38	16.4	Occipital right
4	m	42	Vertigo, Headache	No	Yes	Colloid cyst	Third ventricle	18	1.7	Frontal right
5	f	73	Hakim Trias *	N/A	Yes	Colloid cyst	Third ventricle	9	0.2	Frontal right
6	m	0.5	Vomiting, Ocular motor dysfunction	N/A	Yes	Choroid plexus carcinoma (WHO III)	Lateral ventricle left	30	6.3	Occipital left
7	f	16	Vomiting, Seizures, Somnolence	N/A	Yes	SEGA (WHO I) in TS	Lateral ventricle right	45	23.8	Frontal right
8	m	13	Incidental finding	N/A	Yes	Glioma unclear dignity (m/p LGG)	Thalamic/Foramen of Monro	11	0.6	Frontal right

f = female, m = male, Y = years, D = maximum diameter of the lesion, V = Volume, SEGA = subependymal giant cell astrocytoma, TS = tuberous sclerosis, m/p = most probably, LGG = low grade glioma. * The symptoms consisted of progressive gait insecurity, cognitive impairment with increasing forgetfulness, and new urinary incontinence.

**Table 3:** Outcome measurements of our case series.

No.	PE/EA	EOT	Surgery Time (min)	Blood Loss (mL)	Complications		Neurocognition		mRS		Clinical Symptoms ^‡^	FU	Rec
					Intraop	Postop	Postop	FU	Postop	FU	FU		
1	EA	GTR	150	300	none	Hemianopsia, partial Gerstmann syndrome *	Subjectively normal	Subjectively normal	3	1	1	17.2	no
2	PE	GTR	133	100	none	Cognitive impairment, meningitis	MOCA 18/30	MOCA 24/30	3	2	1	14.7	no
3	EA	GTR	232	250	none	none	Subjectively normal	Subjectively normal	1	0	0	6.8	no
4	PE	GTR	172	50	Transient bleeding, Abrasion of fornix	Cognitive impairment	MOCA 25/30	MOCA 30/30	2	0	0	21.6	no
5	PE	NTR	82	150	none	none	Subjectively normal	Subjectively normal	2	0	0	22.6	no
6	EA	GTR	180	140	none	Secondary hydrocephalus	N/A	N/A	1	1	1	23.2	no
7	EA	GTR	240	100	none	none	N/A	N/A	4 ^†^	3	1	12	no
8	PE	GTR	120	50	Abrasion of fornix	none	Subjectively normal	Subjectively normal	1	1	0	9.3	no

PE = pure endoscopically, EA = endoscopically assisted, EOT = extent of resection, add. I. = additional intervention, MOCA = Montreal cognitive assessment, mRS = modified Rankin scale, FU = follow-up (months), GTR = gross total resection, NTR = near total resection, STR = subtotal resection, CSF = cerebrospinal fluid, EVD = external ventricular drain, VPS = ventriculoperitoneal shunt, N/A = not applicable. * The patient developed postoperatively a partial Gerstmann syndrome with dyscalculia and dysgraphia and a slight hemianopsia. The symptoms were completely regressed at follow-up. ^†^ Patient with mental retardation not able to attend to her own needs; therefore, preoperative and postoperative mRS is high. ^‡^ Evaluation of preoperative clinical symptoms at follow-up with 0 = no symptoms, 1 = improved symptoms, 2 = stable symptoms, and 3 = worsened symptoms.

## Data Availability

The data presented in this study are available on request from the corresponding author.

## Supplementary Materials

The following are available online at https://www.mdpi.com/article/10.3390/jcm10173889/s1, Video: A colloid cyst is completely resected using a purely endoscopic approach via a right frontal burr hole. The OR setup, the equipment, the resection with the NUA (Endoscopic Neurosurgical Pen, Söring GmbH, Quickborn, Germany), and a GTR based on postoperative MRI are shown.
